# Universal Admission Screening for SARS-CoV-2 Infections among Hospitalized Patients, Switzerland, 2020

**DOI:** 10.3201/eid2702.202318

**Published:** 2021-02

**Authors:** Thomas Scheier, Adrian Schibli, Geri Eich, Christian Rüegg, Frank Kube, Adrian Schmid, Urs Karrer, Aline Wolfensberger, Hugo Sax, Peter W. Schreiber

**Affiliations:** University Hospital Zurich, Zurich, Switzerland (T. Scheier, A. Wolfensburger, H. Sax, P.W. Schreiber);; University of Zurich (T. Scheier, A. Wolfensburger, H. Sax, P.W. Schreiber);; City Hospital Triemli, Zurich (A. Schibli, G. Eich);; GZO Wetzikon, Wetzikon, Switzerland (C. Rüegg, F. Kube);; Cantonal Hospital Winterthur, Winterthur, Switzerland (A. Schmid, U. Karrer)

**Keywords:** COVID-19, SARS-CoV-2, epidemiology, emerging infections, asymptomatic transmission, PCR, respiratory infections, severe acute respiratory syndrome coronavirus 2, 2019 novel coronavirus disease, coronavirus disease, zoonoses, viruses, coronavirus, diagnosis, testing, screening, Switzerland

## Abstract

Switzerland began a national lockdown on March 16, 2020, in response to the rapid spread of severe acute respiratory syndrome coronavirus 2 (SARS-CoV-2). We assessed the prevalence of SARS-CoV-2 infection among patients admitted to 4 hospitals in the canton of Zurich, Switzerland, in April 2020. These 4 acute care hospitals screened 2,807 patients, including 2,278 (81.2%) who did not have symptoms of coronavirus disease (COVID-19). Overall, 529 (18.8%) persons had >1 symptom of COVID-19, of whom 60 (11.3%) tested positive for SARS-CoV-2. Eight asymptomatic persons (0.4%) also tested positive for SARS-CoV-2. Our findings indicate that screening on the basis of COVID-19 symptoms, regardless of clinical suspicion, can identify most SARS-CoV-2–positive persons in a low-prevalence setting.

In late 2019, a pneumonia of unknown etiology emerged in Wuhan, Hubei Province, China. In early 2020, public health officials identified the illness as coronavirus disease (COVID-19) and its causative agent as severe acute respiratory syndrome coronavirus 2 (SARS-CoV-2). The World Health Organization declared a pandemic on March 11, 2020 (*1*–*3*), in response to the rapid international spread of COVID-19.

The transmission mode of SARS-CoV-2 is not fully understood; it is thought to be spread mostly by respiratory droplets and direct contact (*4*–*6*). The median incubation period is »5 days (*7*,*8*). Among symptomatic patients, men are affected slightly more frequently than women (*9*,*10*). COVID-19 has many manifestations, ranging from mild upper airway symptoms to acute respiratory distress syndrome. Common signs and symptoms of COVID-19 include fever, cough, sputum production, and fatigue (*11*,*12*). A high proportion of hospitalized COVID-19 patients have concurrent conditions such as arterial hypertension, diabetes mellitus, or coronary heart disease (*13*–*17*).

In March 2020, Rothe et al. published evidence of asymptomatic SARS-CoV-2 transmission (*18*), and evidence that asymptomatic or presymptomatic persons can transmit SARS-CoV-2 infection has continued to increase (*19*,*20*). In many healthcare settings, the number of persons with asymptomatic SARS-CoV-2 infection is unknown. Asymptomatic persons and healthcare workers can contract and spread the infection among hospitalized patients. Many hospitalized patients, who frequently are >65 years of age, have concurrent conditions, or both, are at risk for severe COVID-19.

In consideration of these circumstances, hospitals must take precautions to prevent the spread of SARS-CoV-2. For example, some hospitals might screen patients for SARS-CoV-2 infection within 24 hours before an elective intervention (*21*). Some well-resourced healthcare settings in high incidence areas might benefit from testing patients without COVID-19 symptoms (*22*). In the canton of Zurich, Switzerland, 4 hospitals introduced universal admission screening of all hospitalized patients in April 2020. We used the results of this screening to assess SARS-CoV-2 prevalence among hospitalized patients and to evaluate the additional yield of a universal screening strategy compared to a symptom-driven approach.

## Methods and Materials

### Study Population Characteristics

The canton of Zurich is a region in northeast Switzerland that has a population of »1.5 million inhabitants. The canton has 32 registered hospitals, of which 31 publicly report annual discharge numbers. These 31 hospitals discharged 237,919 patients in 2018 (*23*). During April 1–24, 2020, four hospitals conducted universal admission screening for SARS-CoV-2 (Table 1). The participating sites included the 3 largest hospitals in the canton, which accounted for »44% of discharges in 2018 (Table 1). Screening periods ranged from 11–24 days. The Zurich Cantonal Ethics Commission (Req-2020–00441) waived the requirement for a formal ethical evaluation according to the Swiss Human Research Act.

**Table 1:** Characteristics of 4 hospitals in study on severe acute respiratory syndrome coronavirus 2, canton of Zurich, Switzerland, 2020

Hospital	No. beds	No. patients in 2018	Screening period
GZO Wetzikon	156	10,368	2020 Apr 8–2020 Apr 24
Cantonal Hospital Winterthur	445	27,451	2020 Apr 9–2020 Apr 19
City Hospital Triemli	396	24,335	2020 Apr 8–2020 Apr 24
University Hospital of Zurich	941	41,916	2020 Apr 1–2020 Apr 24

### Testing for SARS-CoV-2

During the screening period, the hospitals tested all patients >16 years of age for SARS-CoV-2 infection, regardless of signs or symptoms. At the time, the health authorities of the canton supported the policy of universal admission screening. Hospital staff informed admitted patients about SARS-CoV-2 testing as a new routine diagnostic procedure. Staff collected a nasopharyngeal swab sample from each patient and tested the samples by PCR. A single laboratory conducted diagnostic procedures for the University Hospital of Zurich (USZ) and GZO Wetzikon (GZO). The other 2 hospitals, City Hospital Triemli (STZ) and Cantonal Hospital Winterthur (KSW), sent samples to separate laboratories. The laboratory that conducted diagnostic procedures for USZ and GZO also tested and confirmed all SARS-CoV-2–positive samples from patients at STZ and a random subset of SARS-CoV-2–positive samples from patients at KSW. PCR methods varied among the participating study sites (Appendix Table 1).

### Symptom Information Collection

Hospital staff assessed each patient for signs and symptoms of COVID-19 at admission. In accordance with guidance provided by the Swiss Federal Office for Public Health, staff considered cough, dyspnea, temperature >38.0°C or feeling feverish, sore throat, and myalgia as possible signs and symptoms of COVID-19 (*24*). The assessment focused on symptoms at the time of the nasopharyngeal sample. Staff also noted whether suspected COVID-19 was the primary reason for admission. Before beginning the study, all participating sites agreed to prospectively collect these variables and document them in medical records. Staff extracted these data from the medical records and entered them into an electronic case report form. When information in the medical chart was inconclusive, we contacted the treating physician or the patient for clarification. At admission, patients were categorized as asymptomatic, (i.e., absence of all COVID-19 signs or symptoms) or symptomatic (i.e., presence of >1 COVID-19 sign or symptom). We compared our results with cantonal data (COVID-19 Informationen Schweiz, https://www.corona-data.ch).

### Statistical Analyses

We analyzed deidentified patient data submitted through an electronic case report form. We conducted statistical analysis using R version 3.3.2 (The R Foundation, https://www.r-project.org). We analyzed the medians and interquartile ranges of continuous variables and frequencies of categorical variables.

## Results

### Incidence of COVID-19

**Figure 1:**
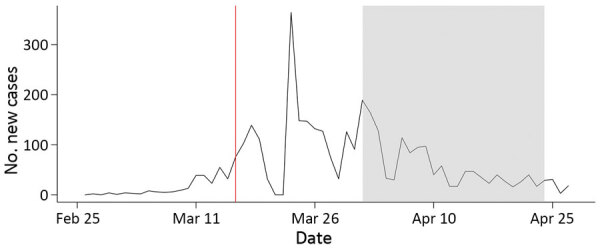
Incidence of severe acute respiratory syndrome coronavirus 2 infection, canton of Zurich, Switzerland, 2020. Data reported as absolute number of daily new diagnosed cases. Red vertical line indicates start of lockdown in Switzerland. Gray shading indicates study period.

In the canton of Zurich, which has »1.5 million inhabitants, the first case of COVID-19 was documented on February 27, 2020 (*25*; Figure 1). The daily incidence of new SARS-CoV-2 infections peaked at 364 cases on March 23, 2020. During the screening period (April 1–24, 2020), the median daily incidence was 40 cases (interquartile range [IQR] 27–87 cases), corresponding to a rate of 2.7 cases/100,000 inhabitants (COVID-19 Informationen Schweiz, https://www.corona-data.ch).

### Study Population

Hospital staff screened 2,807 patients for SARS-CoV-2 infection (Table 2). The median age was 60 years (IQR 39–74 years); 1,368 (48.7%) patients were men and 1,439 (51.3%) women. At admission, 529 (18.8%) patients had >1 sign or symptom of COVID-19: 205 (7.3%) had temperatures >38.0°C or felt feverish, 192 (6.8%) had cough, 282 (10.0%) had dyspnea, 30 (1.1%) had sore throats, and 27 (1.0%) had myalgia. A total of 164 patients (5.8% of the whole study population) were hospitalized primarily for suspected COVID-19.

**Table 2:** Characteristics of hospitalized patients in study on severe acute respiratory syndrome coronavirus 2, canton of Zurich, Switzerland, 2020*

Characteristic	Total	PCR results for severe acute respiratory syndrome coronavirus 2	
		Negative	Positive
Total	2,807	2,739 (97.6)	68 (2.4)
Hospital			
GZO Wetzikon	283	277 (97.9)	6 (2.1)
Cantonal Hospital Winterthur	409	403 (98.5)	6 (1.5)
City Hospital Triemli	583	567 (97.3)	16 (2.7)
University Hospital Zurich	1,532	1,492 (97.4)	40 (2.6)
Median age, y (IQR)	60 (39–74)	60 (39–74)	54.5 (44.5–69)
Sex			
M	1,368	1,330 (97.2)	38 (2.8)
F	1,439	1,409 (97.9)	30 (2.1)
Symptoms			
Any symptom of coronavirus disease	529	469 (88.7)	60 (11.3)
Fever/feeling feverish	205	167 (81.5)	38 (18.5)
Cough	192	152 (79.2)	40 (20.8)
Dyspnea	282	255 (90.4)	27 (9.6)
Sore throat	30	22 (73.3)	8 (26.7)
Myalgia	27	14 (51.9)	13 (48.1)

*Values are no. (%) except as indicated.

### PCR Results

Overall, 68 (2.4%) patients tested positive for SARS-CoV-2 RNA by PCR. Of the 529 patients with >1 sign or symptom of COVID-19, 60 (11.3%) tested positive. In contrast, only 8 (0.4%) of 2,278 patients without symptoms tested positive (Table 2). SARS-CoV-2 infection was diagnosed in 6 (8.8%) patients at GZO, 6 (8.8%) patients at KSW, 16 (23.5%) patients at STZ, and 40 (58.8%) patients at USZ. Asymptomatic SARS-CoV-2–positive patients were identified at all 4 hospitals: 1 (12.5%) patient at GZO, 3 (37.5%) patients at KSW, 1 (12.5%) patient at STZ, and 3 (37.5%) patients at USZ.

**Figure 2:**
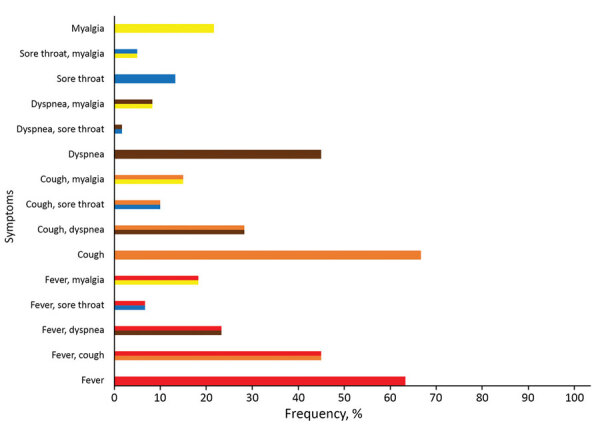
Frequency of >1 symptom of coronavirus disease among patients with symptomatic severe acute respiratory syndrome coronavirus 2 infection, canton of Zurich, Switzerland, 2020. Red indicates fever/feeling feverish; orange indicates cough; brown indicates dyspnea; blue indicates sore throat; yellow indicates myalgia. Unicolor bars indicate 1 symptom; multicolor bars indicate combination of >1 symptoms.

Of the 164 patients admitted primarily for suspected COVID-19, 52 (31.7%) tested positive for SARS-CoV-2 infection by PCR. Of all SARS-CoV-2–infected patients, 38 (55.9%) had temperatures >38.0°C or felt feverish, 40 (58.8%) had cough, 27 (39.7%) had dyspnea, 8 (11.8%) had sore throats, and 13 (19.1%) had myalgia. Among symptomatic COVID-19 patients, the most common manifestations were cough and fever (27; 45%), cough and dyspnea (17; 28.3%), and dyspnea and fever (14; 23.3%) (Figure 2). The absence of COVID-19 signs or symptoms yielded a negative predictive value of 99.6% for SARS-CoV-2–infection.

## Discussion

In this prospective multicenter study, hospital staff tested 2,807 patients, of whom 2,278 (81.2%) did not have signs or symptoms of COVID-19. In total, 68 (2.4%) patients tested positive for SARS-CoV-2 infection by PCR. Of SARS-CoV-2–positive patients, 8 (11.8%) were asymptomatic, corresponding to 0.4% of patients without signs or symptoms of COVID-19. We found that 99.6% of patients without COVID-19 signs or symptoms tested negative for SARS-CoV-2 infection.

On March 16, 2020, the rapid increase in COVID-19 incidence prompted the government of Switzerland to implement and enforce preventive measures including social distancing and the closure of restaurants, bars, entertainment businesses (e.g., cinemas, libraries, museums), and all shops that could not guarantee a minimum distance of 2 meters between persons (*26*). These measures contributed to a sharp decline in COVID-19 incidence. In the canton of Zurich, the incidence of COVID-19 decreased after March 23, 2020, »1 week after the lockdown began in Switzerland. This study started on April 1, 2020, »2 weeks after the beginning of lockdown.

Because of the successful control measures for COVID-19 and the low prevalence of influenza and other respiratory viruses, <20% of the study population had signs or symptoms of COVID-19. Among all persons with SARS-CoV-2 infection, the most frequent symptoms were cough (58.8%) and fever (55.9%), consistent with other reports (*9*,*11*).

We conducted this study because of reports of a large proportion of persons with asymptomatic SARS-CoV-2 infection (*18*,*19*,*27*). In this study, only 0.4% of persons without signs or symptoms of COVID-19 tested positive for SARS-CoV-2 infection. More than 88% of SARS-CoV-2–positive persons had >1 sign or symptom of COVID-19. Our findings are in contrast to Sutton et al. (*28*), who found that 33 (15.4%) of 214 pregnant women tested positive for SARS-CoV-2 infection at time of hospitalization for delivery. Only 4 of those women had COVID-19 symptoms at admission; symptoms developed in 3 more women in the following days. During the study period in New York, NY, USA, from March 22–April 4, a median of 4,958 persons (59 cases/100,000 inhabitants) tested positive for SARS-CoV-2 infection each day (*28*). This rate is »22 times higher than that for the canton of Zurich (median 2.7 cases/100,000 inhabitants) during the period of our study (*29*,*30*). Similarly, Kimball et al. (*31*) reported that 23 (30.3%) of 76 residents in a skilled nursing home tested positive for SARS-CoV-2 infection during a local COVID-19 outbreak. At the time of testing, 13 (56.5%) SARS-CoV-2–positive persons were asymptomatic or had stable, chronic symptoms; COVID-19 symptoms developed in 10 of these 13 previously asymptomatic persons during the 7 days after testing (*31*). The delayed development of symptoms probably indicates that the cases were diagnosed during a presymptomatic period. Another recent study tested for SARS-CoV-2 infection among different subsets of the population in Iceland (*32*). In an open invitation sample of residents of Iceland with no or mild respiratory symptoms (*32*), 87 (0.8%) tested positive for SARS-CoV-2 infection; 36 (41.4%) of these persons were asymptomatic. Researchers also tested a random sample of 2,283 persons living in Iceland, of whom 13 (0.6%) tested positive for SARS-CoV-2 infection; 7 (53.8%) of these persons were asymptomatic. Both sample populations had a 0.3% proportion of persons with asymptomatic SARS-CoV-2 infections (36/10,797 persons in the open invitation sample and 7/2,283 persons in the random sample) (*32*), which is similar to the proportions in our findings (8/2,807 persons; 0.3%). The difference in the proportions of asymptomatic persons among those with SARS-CoV-2 infection (11.8% in this study vs. 41.4% in the open invitation and 53.8% in the random samples from Iceland) (*32*) might have been caused by an overrepresentation of symptomatic persons in our study because COVID-19–compatible symptoms are probably more common among admitted hospital patients.

Identifying and isolating persons with SARS-CoV-2 infection is critical to containing COVID-19. Because of limited testing capacity, healthcare providers must use resources strategically (*33*). Our findings indicate that screening on the basis of COVID-19 symptoms, regardless of clinical suspicion, can identify nearly all SARS-CoV-2–infected persons in the studied epidemiologic setting. Because COVID-19 has a broad spectrum of clinical manifestations, healthcare providers should screen patients for symptoms at admission. By only testing symptomatic patients, healthcare providers can use >80% fewer tests; however, this strategy would not identify 0.4% of SARS-CoV-2 infections. For every »285 persons without symptoms whom we tested, we identified 1 asymptomatic SARS-CoV-2 infection. Whether asymptomatic persons are as infectious as symptomatic persons is unknown. At the time of this study, no quantitative SARS-CoV-2 reporting existed; this lack of data hindered a comparison of viral replication between asymptomatic and symptomatic persons. Additional studies on this topic are urgently needed.

Our study has limitations. First, all participants were tested only once for SARS-CoV-2 infection at admission; no routine follow-up tests were scheduled, regardless of patient signs or symptoms. The lack of follow-up testing might have missed some cases of SARS-CoV-2 infection. Second, we did not collect information on the patients’ potential exposures to SARS-CoV-2 or any other agents of respiratory illness. Third, different laboratories conducted the PCRs with different methods. However, we ensured the validity of results from different laboratories by retesting a subset of SARS-CoV-2–positive samples at another laboratory. Fourth, our findings must be considered in the epidemiologic context; they do not apply to high-prevalence settings with active outbreaks. Fifth, we did not collect information about patients’ COVID-19 symptoms during the previous 2 weeks. We also did not conduct follow-up evaluations of symptoms in patients who were asymptomatic at admission. Further studies should evaluate whether these asymptomatic patients might have been presymptomatic or recovering from COVID-19 at admission. Sixth, we did not collect information on ageusia and anosmia, which were later described as characteristic symptoms among mild and moderate cases of COVID-19 (*34*). Including these variables might have increased the number of symptomatic persons among SARS-CoV-2–infected patients.

This prospective study benefited from a multicenter design and the availability of data on regional incidence of COVID-19. We selected study sites that accounted for »44% of all patient discharges in the canton in 2018 and included the 3 largest hospitals (*23*) and were therefore representative of the canton of Zurich. In addition, no admitted patients refused to participate in the screening for SARS-CoV-2.

In conclusion, universal testing for SARS-CoV-2 of all patients at hospital admission in this region of Switzerland did not identify a substantial number of asymptomatic infections in a low-prevalence setting. Future studies are needed to delineate the role of asymptomatic SARS-CoV-2–infected persons as transmitters in the current pandemic.

Appendix Additional information on universal screening for SARS-CoV-2 among hospitalized patients, Switzerland.

## References

[1] World Health Organization. Rolling updates on coronavirus disease (COVID-19). 2020 [cited 2020 Apr 18]. https://www.who.int/emergencies/diseases/novel-coronavirus-2019/events-as-they-happen

[2] European Centre for Disease Prevention and Control. Rapid risk assessment: Coronavirus disease 2019 (COVID-19) in the EU/EEA and the UK—ninth update. 2020 [cited 2020 Apr 24]. https://www.ecdc.europa.eu/en/publications-data/rapid-risk-assessment-coronavirus-disease-2019-covid-19-pandemic-ninth-update

[3] World Health Organization. Naming the coronavirus disease (COVID-19) and the virus that causes it. 2020 [cited 2020 May 5]. https://www.who.int/emergencies/diseases/novel-coronavirus-2019/technical-guidance/naming-the-coronavirus-disease-(covid-2019)-and-the-virus-that-causes-it

[4] World Health Organization. Modes of transmission of virus causing COVID-19: implications for IPC precaution recommendations. 2020 Mar 29 [cited 2020 Apr 23]. https://www.who.int/news-room/commentaries/detail/modes-of-transmission-of-virus-causing-covid-19-implications-for-ipc-precaution-recommendations

[5] National Center for Immunization and Respiratory Diseases. How COVID-19 spreads. 2020 [cited 2020 May 5]. https://www.cdc.gov/coronavirus/2019-ncov/prevent-getting-sick/how-covid-spreads.html?CDC_AA_refVal=https%3A%2F%2Fwww.cdc.gov%2Fcoronavirus%2F2019-ncov%2Fprepare%2Ftransmission.html

[6] World Health Organization. Report of the WHO-China Joint Mission on Coronavirus Disease 2019 (COVID-19). 2020 Feb [cited 2020 May 5]. https://www.who.int/docs/default-source/coronaviruse/who-china-joint-mission-on-covid-19-final-report.pdf

[7] Ganyani T, Kremer C, Chen D, Torneri A, Faes C, Wallinga J, et al. Estimating the generation interval for coronavirus disease (COVID-19) based on symptom onset data, March 2020. Euro Surveill. 2020;25:2000257. 10.2807/1560-7917.ES.2020.25.17.2000257 32372755 PMC7201952

[8] Lauer SA, Grantz KH, Bi Q, Jones FK, Zheng Q, Meredith HR, et al. The incubation period of coronavirus disease 2019 (COVID-19) from publicly reported confirmed cases: estimation and application. Ann Intern Med. 2020;172:577–82; Epub ahead of print. 10.7326/M20-0504 32150748 PMC7081172

[9] Guan W-J, Ni Z-Y, Hu Y, Liang W-H, Ou C-Q, He J-X, et al.; China Medical Treatment Expert Group for Covid-19. Clinical characteristics of coronavirus disease 2019 in China. N Engl J Med. 2020;382:1708–20. 10.1056/NEJMoa2002032 32109013 PMC7092819

[10] Goyal P, Choi JJ, Pinheiro LC, Schenck EJ, Chen R, Jabri A, et al. Clinical characteristics of COVID-19 in New York City. N Engl J Med. 2020;382:2372–4. 10.1056/NEJMc2010419 32302078 PMC7182018

[11] Wang D, Hu B, Hu C, Zhu F, Liu X, Zhang J, et al. Clinical characteristics of 138 hospitalized patients with 2019 novel coronavirus–infected pneumonia in Wuhan, China. JAMA. 2020;323:1061–9. 10.1001/jama.2020.1585 32031570 PMC7042881

[12] Yang X, Yu Y, Xu J, Shu H, Xia J, Liu H, et al. Clinical course and outcomes of critically ill patients with SARS-CoV-2 pneumonia in Wuhan, China: a single-centered, retrospective, observational study. Lancet Respir Med. 2020;8:475–81; Epub ahead of print. 10.1016/S2213-2600(20)30079-5 32105632 PMC7102538

[13] Zhou F, Yu T, Du R, Fan G, Liu Y, Liu Z, et al. Clinical course and risk factors for mortality of adult inpatients with COVID-19 in Wuhan, China: a retrospective cohort study. [Erratum in: Lancet. 2020;395:1038]. Lancet. 2020;395:1054–62. 10.1016/S0140-6736(20)30566-3 32171076 PMC7270627

[14] Docherty AB, Harrison EM, Green CA, Hardwick HE, Pius R, Norman L, et al.; ISARIC4C investigators. Features of 20 133 UK patients in hospital with covid-19 using the ISARIC WHO Clinical Characterisation Protocol: prospective observational cohort study. BMJ. 2020;369:m1985. 10.1136/bmj.m1985 32444460 PMC7243036

[15] Grasselli G, Zangrillo A, Zanella A, Antonelli M, Cabrini L, Castelli A, et al.; COVID-19 Lombardy ICU Network. Baseline characteristics and outcomes of 1,591 patients infected with SARS-CoV-2 admitted to ICUs of the Lombardy region, Italy. JAMA. 2020;323:1574–81. 10.1001/jama.2020.5394 32250385 PMC7136855

[16] Chow N, Fleming-Dutra K, Gierke R, Hall A, Hughes M, Pilishvili T, et al.; CDC COVID-19 Response Team. Preliminary estimates of the prevalence of selected underlying health conditions among patients with coronavirus disease 2019—United States, February 12–March 28, 2020. MMWR Morb Mortal Wkly Rep. 2020;69:382–6. 10.15585/mmwr.mm6913e2 32240123 PMC7119513

[17] Istituto Superiore di Sanità. Characteristics of SARS-CoV-2 patients dying in Italy: report based on available data on July 22nd, 2020. [cited 2020 Aug 10]. https://www.epicentro.iss.it/en/coronavirus/bollettino/Report-COVID-2019_22_july_2020.pdf

[18] Rothe C, Schunk M, Sothmann P, Bretzel G, Froeschl G, Wallrauch C, et al. Transmission of 2019-nCoV infection from an asymptomatic contact in Germany. N Engl J Med. 2020;382:970–1. 10.1056/NEJMc2001468 32003551 PMC7120970

[19] Furukawa NW, Brooks JT, Sobel J. Evidence supporting transmission of severe acute respiratory syndrome coronavirus 2 while presymptomatic or asymptomatic. Emerg Infect Dis. 2020;26: Epub ahead of print. 10.3201/eid2607.201595 32364890 PMC7323549

[20] Wiersinga WJ, Rhodes A, Cheng AC, Peacock SJ, Prescott HC. Pathophysiology, transmission, diagnosis, and treatment of coronavirus disease 2019 (COVID-19): a review. JAMA. 2020;324:782–93. 10.1001/jama.2020.12839 32648899

[21] Al-Muharraqi MA. Testing recommendation for COVID-19 (SARS-CoV-2) in patients planned for surgery - continuing the service and ‘suppressing’ the pandemic. Br J Oral Maxillofac Surg. 2020;58:503–5. 10.1016/j.bjoms.2020.04.014 32307131 PMC7152878

[22] European Society of Cardiology. ESC Guidance for the Diagnosis and Management of CV Disease during the COVID-19 Pandemic. 2020 [cited 2020 May 11]. https://www.escardio.org/Education/COVID-19-and-Cardiology/ESC-COVID-19-Guidance

[23] Kanton Zürich Gesundheitsdirektion. Healthcare 2019 acute somatics rehabilitation psychiatry [in German]. 2020 [cited 2020 Aug 10]. https://www.zh.ch/content/dam/zhweb/bilder-dokumente/themen/gesundheit/gesundheitsversorgung/spitaeler_kliniken/daten_und_statistik_der_listenspitaeler/gesundheitsversorgungsbericht/gesundheitsversorgungsbericht_2019.pdf

[24] Bundesamt für Gesundheit. COVID-19: Empfehlungen zum Umgang mit erkrankten Personen und Kontakten ab 19. Bern (Switzerland): Eidgenössisches Departement des Innern; 2020.

[25] Kanton Zürich. Coronavirus: first case in the Canton of Zurich [in German]. 2020 Feb 27 [cited 2020 Nov 11]. https://www.zh.ch/de/news-uebersicht/medienmitteilungen/2020/02/coronavirus-erster-fall-im-kanton-zuerich.html

[26] Der Bundesrat. Coronavirus: federal council declares the extraordinary situation and tightened the measures [in German]. 2020 Mar 17 [cited 2020 May 4]. https://www.admin.ch/gov/de/start/dokumentation/medienmitteilungen.msg-id-78454.html

[27] Arons MM, Hatfield KM, Reddy SC, Kimball A, James A, Jacobs JR, et al.; Public Health–Seattle and King County and CDC COVID-19 Investigation Team. Presymptomatic SARS-CoV-2 infections and transmission in a skilled nursing facility. N Engl J Med. 2020;382:2081–90. 10.1056/NEJMoa2008457 32329971 PMC7200056

[28] Sutton D, Fuchs K, D’Alton M, Goffman D. Universal screening for SARS-CoV-2 in women admitted for delivery. N Engl J Med. 2020;382:2163–4. 10.1056/NEJMc2009316 32283004 PMC7175422

[29] New York City Department of Health and Mental Hygiene. COVID-19: data. 2020 [cited 2020 May 5]. https://www1.nyc.gov/site/doh/covid/covid-19-data.page#download

[30] New York City Department of City Planning. Population—current and projected populations. 2018 [cited 2020 Apr 28]. https://www1.nyc.gov/site/planning/planning-level/nyc-population/current-future-populations.page

[31] Kimball A, Hatfield KM, Arons M, James A, Taylor J, Spicer K, et al.; Public Health – Seattle & King County; CDC COVID-19 Investigation Team. Asymptomatic and presymptomatic SARS-CoV-2 infections in residents of a long-term care skilled nursing facility—King County, Washington, March 2020. MMWR Morb Mortal Wkly Rep. 2020;69:377–81. 10.15585/mmwr.mm6913e1 32240128 PMC7119514

[32] Gudbjartsson DF, Helgason A, Jonsson H, Magnusson OT, Melsted P, Norddahl GL, et al. Spread of SARS-CoV-2 in the Icelandic population. N Engl J Med. 2020;382:2302–15. 10.1056/NEJMoa2006100 32289214 PMC7175425

[33] World Health Organization. Laboratory testing strategy recommendations for COVID-19. 2020 [cited 2020 May 4]. https://apps.who.int/iris/bitstream/handle/10665/331509/WHO-COVID-19-lab_testing-2020.1-eng.pdf

[34] Lechien JR, Chiesa-Estomba CM, De Siati DR, Horoi M, Le Bon SD, Rodriguez A, et al. Olfactory and gustatory dysfunctions as a clinical presentation of mild-to-moderate forms of the coronavirus disease (COVID-19): a multicenter European study. Eur Arch Otorhinolaryngol. 2020;277:2251–61. 10.1007/s00405-020-05965-1 32253535 PMC7134551

