# Supplementary material for: Universal Admission Screening for SARS-CoV-2 Infections among Hospitalized Patients, Switzerland, 2020
Source: Emerg Infect Dis. 2021 Feb;27(2):404–10. doi: 10.3201/eid2702.202318 (PMC7853575; doi:10.3201/eid2702.202318)
Supplement: Appendix — Additional information on universal screening for SARS-CoV-2 among hospitalized patients, Switzerland. [file 20-2318-Techapp-s1.pdf]

Article DOI: https://doi.org/10.3201/eid2702.202318

Universal Admission Screening for SARS-CoV-2 Infection among Hospitalized Patients, Switzerland, 2020

Appendix

Appendix Table. PCRs used to detect SARS-CoV-2, Switzerland, 2020

Hospital	PCR	Target
GZO Wetzikon	In-house assay according to V.M. Corman et al. (*1*) or<br>Roche cobas SARS-CoV-2 IVD test	open reading frame 1, envelope protein gene<br><br>open reading frame 1, envelope protein gene
Cantonal Hospital Winterthur*	In-house assay using TIB MOLBIOL LightMix Modular Sarbecovirus E-gene and Wuhan CoV RdRp-gene (TIB MOLBIOL, https://www.tib-molbiol.com)	envelope protein gene, RNA-dependent RNA polymerase gene
City Hospital Triemli†	CDC 2019-Novel Coronavirus (2019-nCoV) Real-Time RT-PCR Diagnostic Panel (*2*)	Viral nucleocapsids 1 and 2, RNase P
University Hospital of Zurich	In-house assay according to V.M. Corman et al. (*1*) or<br>Roche cobas SARS-CoV-2 IVD test	open reading frame 1, envelope protein gene<br><br>open reading frame, 1 envelope protein gene

*A subset of SARS-CoV-2–positive samples was confirmed by identical PCR at GZO Wetzikon and University Hospital of Zurich. E, envelope protein; ORF, open reading frame; SARS-CoV-2, severe acute respiratory syndrome coronavirus 2.
†All SARS-CoV-2–positive samples were reanalyzed by the laboratory performing SARS-CoV-2 diagnostics for the University Hospital of Zurich and GZO Wetzikon with an in-house assay according to V.M. Corman et al. (*1*) or Roche cobas SARS-CoV-2 IVD test (Roche Diagnostics, https://diagnostics.roche.com).

References

1. Corman VM, Landt O, Kaiser M, Molenkamp R, Meijer A, Chu DK, et al. Detection of 2019 novel coronavirus (2019-nCoV) by real-time RT-PCR. Euro Surveill. 2020;25:2000045. PubMed https://doi.org/10.2807/1560-7917.ES.2020.25.3.2000045

2. Centers for Disease Control and Prevention, CDC 2019-novel coronavirus (2019-nCoV) real-time RT-PCR diagnostic panel. 2020 Jul 13 [cited 2020 Aug 19]. https://www.fda.gov/media/134922/download
